# Tumor Suppressor Candidate 2 (TUSC2): Discovery, Functions, and Cancer Therapy

**DOI:** 10.3390/cancers15092455

**Published:** 2023-04-25

**Authors:** Austin Arrigo, Angelina T. Regua, Mariana K. Najjar, Hui-Wen Lo

**Affiliations:** 1 Department of Neurosurgery, McGovern Medical School, University of Texas Health Science Center at Houston, Houston, TX 77030, USAangelina.t.regua@uth.tmc.edu (A.T.R.); mariana.k.najjar@uth.tmc.edu (M.K.N.); 2 Graduate School of Arts and Sciences, Wake Forest University, Winston-Salem, NC 27101, USA

**Keywords:** N-terminal myristoylation, apoptosis, tumor suppressor, mitochondrial calcium homeostasis, TUSC2, cancer

## Abstract

**Simple Summary:**

Tumor Suppressor Candidate 2 (TUSC2) is an important tumor suppressor that negatively regulates cancer growth and progression in multiple cancer types. TUSC2 was first discovered as a candidate tumor suppressor gene that is located on a frequently deleted region of chromosome 3p21.3 in lung cancer. Since its initial discovery in lung cancer, TUSC2 loss has also been reported in glioma, sarcoma, and cancers of the breast, ovaries, and thyroid. When TUSC2 is re-expressed in these cancer types, it reduces cancer cell growth and promotes cell death. In normal cells, TUSC2 regulates the immune system and prevents the development of autoimmune diseases. TUSC2 also regulates calcium movement within the cell and the mitochondria, which is important for overall cellular function. Lastly, TUSC2 loss promotes premature aging with the development of hearing loss and sporadic Alzheimer’s disease. Altogether, these findings define a vital role that TUSC2 plays in normal and cancerous cells.

**Abstract:**

Tumor Suppressor Candidate 2 (TUSC2) was first discovered as a potential tumor suppressor gene residing in the frequently deleted 3p21.3 chromosomal region. Since its discovery, TUSC2 has been found to play vital roles in normal immune function, and TUSC2 loss is associated with the development of autoimmune diseases as well as impaired responses within the innate immune system. TUSC2 also plays a vital role in regulating normal cellular mitochondrial calcium movement and homeostasis. Moreover, TUSC2 serves as an important factor in premature aging. In addition to TUSC2′s normal cellular functions, TUSC2 has been studied as a tumor suppressor gene that is frequently deleted or lost in a multitude of cancers, including glioma, sarcoma, and cancers of the lung, breast, ovaries, and thyroid. TUSC2 is frequently lost in cancer due to somatic deletion within the 3p21.3 region, transcriptional inactivation via TUSC2 promoter methylation, post-transcriptional regulation via microRNAs, and post-translational regulation via polyubiquitination and proteasomal degradation. Additionally, restoration of TUSC2 expression promotes tumor suppression, eventuating in decreased cell proliferation, stemness, and tumor growth, as well as increased apoptosis. Consequently, TUSC2 gene therapy has been tested in patients with non-small cell lung cancer. This review will focus on the current understanding of TUSC2 functions in both normal and cancerous tissues, mechanisms of TUSC2 loss, TUSC2 cancer therapeutics, open questions, and future directions.

## 1. Introduction

Tumor Suppressor Candidate 2 (*TUSC2*/*FUS1*) was first discovered as a candidate tumor suppressor gene (TSG) within the 630-kb homozygous deletion on chromosomal region 3p21.3 in lung cancer [1]. This chromosomal region was first identified as one of the four allele regions lost on chromosomal region 3p in both small cell lung cancer (SCLC) and non-small cell lung cancer (NSCLC) cases [2,3,4]. The 3p21.3 region was studied specifically for its frequent deletion in preneoplastic lung cancer lesions in current and former smokers, suggesting that the 3p21.3 region contains a cluster of TSGs whose loss contributes to lung cancer initiation [2,3,5,6]. Further analysis narrowed the region of interest to a critical gene region of 370 kb, and even further to a 120 kb region most likely containing critical TSGs deleted in both breast and lung cancer [7,8]. Through generation of multiple cosmid cloning vectors of the 630-kb clone region followed by Sanger sequencing, Lerman et al. discovered 25 resident genes and multiple candidate genes in the 3p21.3 region, with 10 genes located in the 370 kb segment of the critical gene region, and 9 within the 120 kb segment of the same region. Of these 25 resident genes, they determined that *TUSC2/FUS1* is one of the potential TSGs located within the 120 kb segment of the 370 kb critical gene region. *TUSC2/FUS1* (herein referred to as *TUSC2*) was named for its positioning as the “fusion” junction between cosmid LUCA12 and LUCA13 [1]. 

*TUSC2* is a 3.3 kb gene that contains three exons, which are transcribed into a 1.8 kb mRNA transcript that is abundantly expressed in all human tissues [1]. Translation of *TUSC2* mRNA produces a 110 amino acid protein consisting of multiple functional domains including a myristoylation motif (1–9 aa), myristoyl binding motif (45–110 aa), and an EF-hand calcium-binding motif (54–66 aa) [1,9,10]. Two post-translational modifications (PTMs) are frequently found on TUSC2, including N-terminal myristoylation and poly-ubiquitination of TUSC2 lysine residues K71, K84, and K93 [9,11]. TUSC2 N-terminal myristoylation covalently adds a 14-carbon myristoyl group to the N-terminal glycine residue (G2) during protein translation [9,12]. N-terminal myristoylated proteins have critical biological functions, including calcium and ion-channel regulation, controlling protein localization and altering cellular structure and the regulation of protein–protein and protein–substrate interactions [13,14,15]. Uno et al. found N-terminally myristoylated TUSC2 to induce apoptosis, suppress cell growth, and suppress tumor xenografts and lung metastasis in vivo. Conversely, unmyristoylated TUSC2 had significantly reduced tumor suppressive function, resulting in reduced TUSC2 mediated apoptosis and cell cycle inhibition in lung cancer. Moreover, they found unmyristoylated TUSC2 to be more susceptible to proteasomal degradation in lung cancer. Furthermore, they generated a myristoylation deficient TUSC2 mutant, Myr-mut-FUS1, which resulted in a shorter duration of transient protein expression and had a protein half-life of 6 h compared to the 12 h of wt-FUS1 (wild type TUSC2). Additionally, Myr-mut-FUS1 protein half-life was stabilized and comparable to wt-FUS1 upon inhibition of the proteasome, demonstrating that FUS1 (TUSC2) protein stability is regulated through the ubiquitin proteasomal degradation system [9]. Proteins are most commonly marked for proteasomal degradation via protein polyubiquitination [16]. TUSC2 is poly-ubiquitinated on lysine residues K71, K84, and K93 in glioblastoma (GBM) [11]. We recently reported that poly-ubiquitination of TUSC2-K71 significantly decreases TUSC2 protein stability. Conversely, we found that point mutation of this TUSC2 residue (TUSC2-K71R) decreased TUSC2 poly-ubiquitination, and results in enhanced TUSC2 protein stability, indicating that TUSC2 protein stability is, in part, regulated through TUSC2-K71 polyubiquitination and subsequent TUSC2 proteasomal degradation [11]. 

Currently, the overall functions of TUSC2 both in normal and cancerous tissues are still not well understood. Recent discoveries determined that TUSC2 has multiple roles in overall normal cellular homeostasis, though the mechanisms of how TUSC2 regulates normal cellular functions remain unclear. Additionally, TUSC2 loss is studied in multiple cancers, including lung, breast, thyroid, ovarian, mesothelioma, gliomas, and other cancer types [9,11,17,18,19,20]. This review will focus on the known functions of TUSC2 in normal tissues and the tumor suppressive functions of TUSC2 in multiple cancer types, as well as efforts in developing anti-cancer therapies that focus on restoring or sustaining TUSC2 expression.

## 2. TUSC2 Normal Cellular Functions

### 2.1. TUSC2 and Calcium Regulation

**Figure 1:**
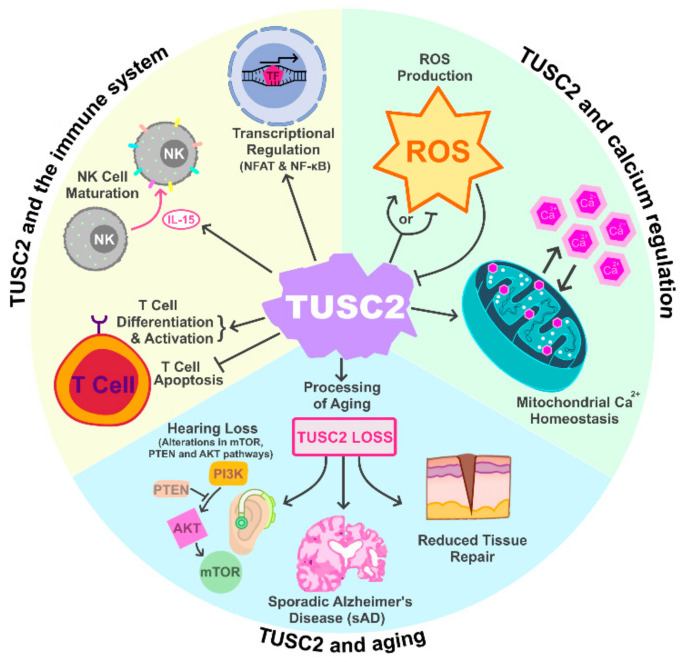
TUSC2 Normal Cellular Functions. TUSC2 has been shown to play important roles in regulating the immune system, cellular calcium regulation as well as the process of aging.

TUSC2 is a major regulator of mitochondrial calcium handling and intracellular calcium homeostasis (Figure 1) [10]. The TUSC2 amino acid sequence contains a highly homologous region to an EF-hand calcium binding domain, spanning from amino acids 54 to 65 [10,21]. The EF-hand motif is the most common calcium-binding motif found in calcium binding proteins and is found in more than 66 subfamilies of calcium-binding proteins, including the proteins calmodulin and troponin [13,22,23,24]. Calmodulin and troponin are EF-hand calcium-binding proteins that mediate calcium (Ca^2+^) movement for intracellular calcium dependent signaling pathways and cardiac and skeletal muscle contraction respectively [21,22,23]. Calcium ions are involved in a multitude of cellular processes, including altering protein conformation, muscle contraction, cell proliferation, cell development, and cell death [25,26,27]. Ca^2+^ is also important for overall mitochondrial function and regulation [28,29]. Ca^2+^ influx can lead to mitochondrial membrane depolarization, or it can be used as a buffer for overall cellular Ca^2+^ homeostasis [28]. Recoverin is one such calcium-binding protein that contains multiple EF-hand calcium binding motifs, an N-terminal myristoylation site, and is classified as a calcium/myristoyl switch protein [13,14,30,31,32]. Upon binding of Ca^2+^ to two of the four EF-hand motifs on recoverin, N-terminal myristoylated recoverin “switches” and releases the hydrophobic myristoyl group from its myristoyl binding pocket, which anchors recoverin into cellular membranes [13,33]. Sequence and protein homology analyses determined that TUSC2 shares the highest homology with the recoverin protein, with TUSC2 sharing 53% homology to the myristoyl binding domain of recoverin [10]. Due to its homology to recoverin, as well as the identification of its N-terminal myristoylation and EF-hand motif, TUSC2 can be classified as a calcium/myristoyl switch protein [9,10,13,14,30]. Additionally, the N-terminal myristoylation is important for TUSC2 subcellular membrane localization, specifically in the mitochondria [9,10].

TUSC2 is also important for regulating mitochondrial calcium homeostasis [10,34]. Uzhachenko et al. illustrated that TUSC2 knockout (TUSC2-KO) elevates mitochondrial calcium levels and disrupts the ability of mitochondria to properly accumulate calcium following treatment with ionomycin, an ionophore that binds free floating calcium, in epithelial and immune cells. This dysregulation of mitochondrial calcium accumulation results from excessive Ca^2+^ efflux from the mitochondrial Na^+^/Ca^2+^ exchanger following TUSC2-KO [10,35]. Furthermore, TUSC2 loss and altered mitochondrial calcium regulation result in increased mitochondrial fission and fragmentation, which signifies mitochondrial stress [10,36]. 

Mitochondrial calcium homeostasis is important in regulating overall mitochondrial function and for regulating the formation of reactive oxygen species (ROS) [37]. Interestingly, TUSC2 expression has been shown to be downregulated by ROS, and ROS levels has been found to be regulated by TUSC2, suggesting a potential feedback or regulatory loop between TUSC2 and mitochondrial ROS production [17,34,38]. Whether TUSC2 promotes or inhibits ROS production is dependent on cell type. For example, in innate immune TUSC2-KO cell populations, TUSC2 loss results in a significant decrease in ROS production, whereas ROS production was significantly elevated in TUSC2-KO B and T cells, suggesting that TUSC2 regulates mitochondrial ROS homeostasis [34]. Additionally, TUSC2 loss has been found to increases ROS production in multiple cancer lines [34]. Elevated ROS levels influence multiple stages of cancer progression, including tumorigenesis, metastasis, and cell survival [39,40,41]. However, high levels of ROS may promote cancer cell apoptosis [42]. Together, these studies demonstrate the importance of TUSC2 mitochondrial functions in relation to maintaining the overall mitochondrial calcium homeostasis within the cell.

### 2.2. TUSC2 and the Immune System

A previous report by Ivanova et al. demonstrated that mice with conventional TUSC2-KO exhibited altered overall immune responses, suggesting that TUSC2 may regulate processes within the immune system (Figure 1) [43]. Ivanova and colleagues also found that mice with TUSC2 heterozygous and TUSC2 homozygous KO displayed greater mortality between the ages of two and 14 months compared to their wild-type littermates, with 11% of either heterozygous or homozygous TUSC2-KO cohorts dying or reaching humane endpoints. Deceased TUSC2-KO mice frequently showed signs of systemic infection, fibrinoid necrotizing arteritis in multiple organs, severe nephropathy, anemia, and glomerulonephritis, which are pathologies associated with autoimmune disorders such as systemic lupus erythematosus [44,45]. Additionally, deceased TUSC2-KO mice frequently exhibited spontaneous tumors, including hemangioma, hemangiosarcoma, lymphoma, and other spontaneous malignancies, indicating that TUSC2 loss may play a role in tumorigenesis. At two years of age, 23% of TUSC2 heterozygous KO and 20% of TUSC2 homozygous KO mice had multi-organ arteritis compared to WT mice. Additionally, TUSC2-KO mice also had high levels of circulating autoreacting nuclear antibodies, further implicating TUSC2 as an important regulator of the immune system and TUSC2 loss as a potential driver of autoimmune disorder development [43]. 

In addition to regulating autoimmune disorder development, TUSC2 has been implicated in the immune response and inflammation in an asbestos-mesothelioma TUSC2-KO mouse study [34]. Uzhachenko et al. demonstrated that TUSC2 loss is associated with an increased inflammatory response following injection of asbestos, a well-documented irritant that promotes inflammation, into TUSC2-KO mice. Conventional TUSC2-KO mice displayed reduced spleen and liver mass as well as altered protein accumulation in the peritoneal fluid following asbestos injection, demonstrating an overall altered immune response compared to TUSC2-WT mice. Furthermore, histopathological analyses revealed that TUSC2-KO mice exposed to asbestos had inflamed mesenteric fat with prominent infiltration of fibroblasts and Langhans-type giant cells, as well as increased positive Ki-67 staining in the mesothelial cells, demonstrating signs of chronic inflammation. TUSC2-KO mice also demonstrated a significant increase in pro-inflammatory cytokines *IFNγ* and *IL-1α,* with a significant decrease in regulatory cytokines such as *IL-10*. Reduced T cell activation, as well as altered ROS levels and mitochondrial membrane potential in innate and adaptive immune cells, are also reported as a result of TUSC2-KO in mice, further demonstrating the importance of TUSC2 in immune cell function [34]. 

TUSC2 is highly expressed in human T and B cells, with increased expression in activated T cells [43]. TUSC2 regulates T cell activation and differentiation by promoting expression of the vital T cell surface markers CD4, PD-1, and PD-L1 [10,46]. Conversely, TUSC2 loss significantly decreases the surface expression of T cell surface markers, thereby reducing T cell activation and differentiation [10]. Concordantly, TUSC2 restoration in lung cancer cell lines decreased PD-L1 expression, promoting an anti-tumor immune response and enhancing the efficacy of anti-PD-1 and anti-PD-L1 therapies [47,48,49,50]. Alternatively, TUSC2 loss leads to impaired mitochondrial calcium handling and results in the downregulation of many genes required for T cell activation via the CD3/CD28 receptor pathway, including *TNF-α, IRF4,* and *IL-2* [10]. Furthermore, TUSC2 inhibits T cell proliferation while having no effect on T cell apoptosis, and is hypothesized to inhibit Th1 differentiation following CD3/CD28 stimulation, which strongly suggests that TUSC2 regulates overall T cell function [10].

TUSC2 also promotes natural killer (NK) cell maturation [43]. In the conventional TUSC2-KO mouse model generated by Ivanova et al., TUSC2-KO mice had a 45% decrease in total NK cell numbers and a 57% decrease in their percentage of mature NK cells as compared to WT mice. Moreover, TUSC2-KO mice had significantly lower levels of the cytokine IL-15, a critical cytokine for NK cell maturation [43]. Treatment with IL-15 restored the mature NK cell population in TUSC2-KO mice, indicating that IL-15 may be an important factor downstream of TUSC2 for promoting NK cell maturation [17,43]. IL-15 expression was later found to be significantly increased following TUSC2 overexpression, supporting the role of TUSC2 in regulating IL-15 expression and the importance of TUSC2 in NK cell maturation [17].

In addition to IL-15, TUSC2 regulates the expression of multiple immune-related genes [10,17,34,46]. TUSC2 overexpression in mesothelioma cells altered expression of 46 immune-related genes, whereas the expression of *IFNγ, IFNγR, IL-1α, IL-1β, CCL5,* and *IL-10* was significantly increased in immune cells derived from a conventional TUSC2-KO mouse model [17,34]. These findings indicate that TUSC2 mediated immune regulation may arise from altered expression of multiple downstream immune related genes [17,34]. Additionally, Uzhachenko et al. discovered that TUSC2 regulates the calcium-dependent transcription factor NFAT (nuclear factor of activated T-cells) and NF-κB (nuclear factor kappa-light-chain-enhancer of activated B cells), which are transcription factors that regulate inflammation [10]. Genes suppressed by TUSC2 in T-cells have promoters that are enriched in binding sites for both NFAT and NF-κB, as well as binding sites for NFAT-cooperating transcription factors MAF, IRF, and OCT1 [10]. Furthermore, many of the genes upregulated in TUSC2-KO T cells, such as *resistin* (which promotes NF-κB activation) and *S100a8/S100a9,* which are key genes in promoting the inflammation associated with rheumatoid arthritis and inflammatory bowel disease, are pro-inflammatory [10,51]. Many downstream genes regulated by TUSC2 and NFAT/NF-κB are involved in extracellular matrix remodeling or overall cellular calcium handling, suggesting that the TUSC2/NFAT/NF-κB pathway may regulate important calcium controlled immune functions [10]. Interestingly, TUSC2 also regulates the NF-κB pathway in osteoclasts through increasing the activation of receptor activator of nuclear factor κB ligand (RANKL), promoting bone reabsorption [46]. Overall, TUSC2 functions as an important factor involved in overall immune system regulation through multiple downstream immune related genes, specifically in T, B, and NK cells, controlling the innate immune response and the development of autoimmune disorders. 

### 2.3. TUSC2 and Aging

Studies have also found TUSC2 to be involved in the processes of aging (Figure 1). In a conventional TUSC2-KO mouse model, Coronas-Somano et al. found that TUSC2 loss induced sporadic Alzheimer’s Disease (sAD) in mice. TUSC2-KO mice presented with multiple cellular alterations associated with cognitive and olfactory sAD symptoms, such as disrupted mitochondrial homeostasis with increased oxidative stress and altered calcium signaling, as well as increased autophagy within the brain. Along with signaling pathway alterations, TUSC2-KO mice demonstrated deficits in olfactory and spatial memory and showed longer sleep times during the diurnal cycle [52]. In a follow up study, Uzhachenko et al. found that TUSC2-KO mice also presented with multiple symptoms of premature aging such as lordokyphosis, which is an arched or curved spine; reduced stress tolerance; lack of vigor; and premature death. TUSC2-KO mice also presented with low sperm count, chronic inflammation, as well as a reduced ability to repair tissue damage. The mitochondria of TUSC2-KO mice showed altered dynamics in calcium accumulation and had an overall lower ability to produce energy quickly [53]. Additionally, Tan and colleagues also found TUSC2 loss to be associated with age-related hearing loss resulting from changes in cellular metabolism. The hearing loss is attributed to alterations in the antioxidant and nutrient and energy sensing pathways of mTOR, PTEN, and AKT within the cochleae of the TUSC2-KO mice. Interestingly, treatment of TUSC2-KO mice with an antioxidant treatment, N-acetyl cysteine, resulted in molecular changes that restored hearing responses, mitochondrial structure, and delayed the onset of hearing loss in TUSC2-KO mice [54]. A similar TUSC2-KO mouse study demonstrated a role for TUSC2 in radioprotection [55]. Yazlovitskaya et al. showed that TUSC2-KO mice had higher mortality rates following radiation as compared to the TUSC2-KO mice. Moreover, GI crypt epithelial cells of the TUSC2-KO mice were more apoptotic and exhibited dysregulated cellular pathways involved in immune response, oxidative stress response, apoptosis, cell cycle, and DNA repair [55]. Thus, TUSC2 loss is associated with multiple phenotypes of premature aging, including sAD, reduced tissue repair, hearing loss, altered antioxidant and nutrient and energy signaling pathways, and radiosensitivity in TUSC2-KO mice.

## 3. TUSC2 in Cancers

### 3.1. Lung Cancer

Lung cancer is the second most frequently diagnosed cancer and is the leading cause of cancer related deaths in the world, with nearly 240,000 new cases and 130,000 deaths estimated in the United States alone in 2023 [56]. Lung cancer is classified as SCLC or NSCLC based on the cancerous cell of origin [57]. NSCLC can be further classified into adenocarcinoma and squamous cell carcinoma [57,58]. Standard of care therapy for both NSCLC and SCLC consists of surgery, radiation therapy, and targeted therapies [59,60].

**Figure 2:**
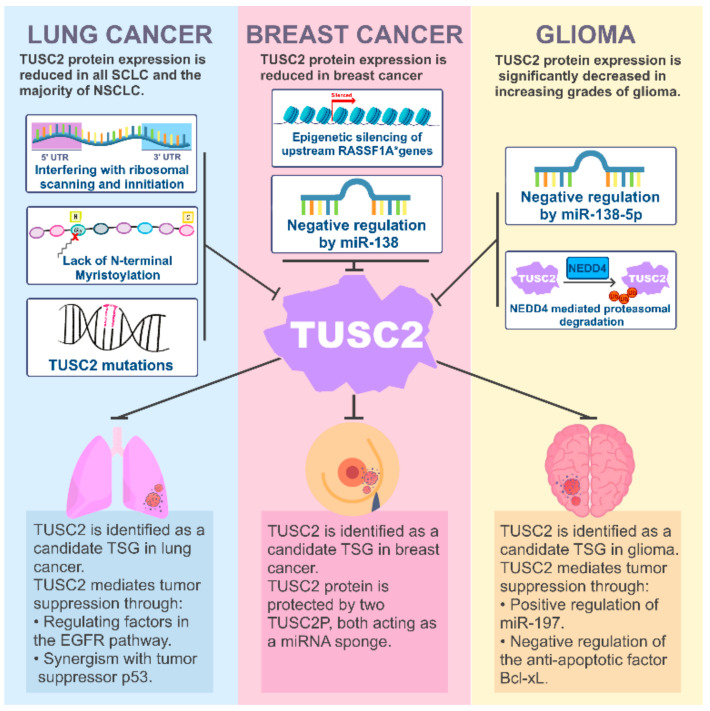
TUSC2 in Cancer. TUSC2 is a tumor suppressor lost in multiple types of cancer, including lung, breast, and gliomas. TUSC2 overexpression leads to tumor apoptosis, and decreased tumor growth, whereas TUSC2 loss promotes a more aggressive cancer phenotype across multiple cancer types.

TUSC2 protein is expressed in normal human bronchial epithelial cells and fibroblasts but is markedly reduced in all SCLC and the majority of NSCLC cases [9,61,62] (Figure 2). Interestingly, although TUSC2 protein expression is reduced in the majority of lung cancer samples, TUSC2 mRNA is expressed in the majority of lung cancer samples, with less than 20% of patients having TUSC2 promoter methylation, suggesting that TUSC2 expression is post-transcriptionally altered in lung cancers [61,63]. Loss of TUSC2 protein expression in lung cancer may be mediated through the 5′ and 3′ untranslated regions (UTR) of the TUSC2 mRNA [64,65]. Indeed, two upstream open-reading frames in the 5′ UTR of TUSC2 mRNA were shown to interfere with ribosomal scanning and the initiation of protein translation [64]. Additionally, multiple key elements were found within the 3′ UTR that lead to altered TUSC2 mRNA regulation in lung cancer, resulting in reduced TUSC2 protein expression [64]. MiRNA target prediction software PicTar, TargetScan, MiRanda, miGTS, and miRmate found miR-19a, miR-378, miR-93, miR-98, and miR-197 to be the most likely miRNAs targeting the 3′ UTR elements of TUSC2 [65,66,67,68,69]. MiR-19a, miR-378, miR-93, miR-98, and miR-197 are all highly expressed in lung cancer patient samples and cell lines and promote lung cancer cell survival and growth through the downregulation of TUSC2. However, TUSC2 overexpression abrogates the pro-tumorigenic roles of these miRNAs [65,66,67,68,69,70].

In addition to miRNA-mediated TUSC2 downregulation, TUSC2 N-terminal myristoylation also regulates TUSC2 protein expression in lung cancer [9]. Non-myristoylated TUSC2 is not found in normal lung tissue. However, myristoylated and non-myristoylated TUSC2 are detected in lung cancer samples [9]. Non-myristoylated TUSC2 is targeted for proteasomal degradation, resulting in protein instability and a shorter TUSC2 protein half-life [9]. Non-myristoylated TUSC2 displays an impaired ability to impede colony formation in vitro and lung cancer growth in vivo compared to N-terminal myristoylated TUSC2, suggesting that myristoylation of TUSC2 is required, in part, for its tumor suppressor function [9]. Interestingly, TUSC2 is infrequently mutated in lung cancer patients, with only 5% of lung cancer cases presenting with a TUSC2 mutation [1]. The three known TUSC2 mutations are non-sense mutations resulting in truncated forms of TUSC2 [1]. A fourth non-sense mutation was later discovered as a result of aberrant TUSC2 mRNA splicing that produced a 28 nucleotide loss at the 3′ terminus of exon 2 [61]. This splicing results in the formation of an 82 amino acid truncated TUSC2 [61]. The effect of these mutation on TUSC2 function has not been investigated in lung cancer [1,61].

Multiple studies have demonstrated that TUSC2 is highly tumor suppressive in lung cancer [9,61,71,72,73]. TUSC2 overexpression in H1299 lung cancer cells significantly decreases colony formation and induces G1 cell cycle arrest [9,61,72]. Furthermore, injection of a TUSC2 plasmid complexed in a N-[1-(2,3-dioleoyloxy)propyl]-N,N,N-trimethylammonium chloride (DOTAP):cholesterol liposomes resulted in decreased primary tumor growth, decreased metastatic nodules, increased tumor apoptosis, and promoted survival in a lung cancer xenograft mouse model [71,74].

One potential mechanism for TUSC2-mediated tumor suppression in lung cancer may occur through TUSC2 regulation of the epidermal growth factor receptor (EGFR) pathway. Restoration of TUSC2 expression in lung cancer cells significantly inhibits tumor cell growth and colony formation and increases the sensitivity of cultured resistant lung cancer cells to the EGFR inhibitor erlotinib [73,75]. TUSC2 overexpression and erlotinib treatment demonstrate an additive effect, suppressing NSCLC both in vitro and in vivo, and co-administration of TUSC2 and erlotinib decreased tumor growth and metastasis, and increased apoptosis compared to controls or single agent treatment [75]. Additionally, TUSC2 regulates multiple downstream EGFR targets, such as FGFR2, mTOR, AKT, and c-Abl in lung cancer [72,75,76,77]. TUSC2 alone and TUSC2 in combination with erlotinib significantly decreases expression of FGFR2 tyrosine kinase, a known driver of EGFR inhibitor resistance [75,77]. Additionally, TUSC2 overexpression significantly decreases mTOR phosphorylation and activity, which are further inhibited following administration of erlotinib [75]. TUSC2 overexpression also sensitized multiple lung cancer cell lines to treatment with the AKT inhibitor MK2206 in vitro and in vivo [72]. TUSC2 overexpression significantly increased phosphorylation and enzymatic activity of AMP-activated protein kinase (AMPK), which is necessary for TUSC2-mediated MK2206 sensitivity in lung cancer cells [72]. Interestingly, TUSC2 directly interacts with and inhibits the activity of c-Abl tyrosine kinase, which provides an alternative mechanism of TUSC2-mediated suppression of lung cancer [76]. Furthermore, TUSC2 expression and erlotinib treatment increase intracellular ROS, promoting cell death, and further addition of the thioredoxin reductase 1 inhibitor, auranofin, further increases cellular ROS, inhibits colony formation, and increases lung cancer apoptosis, suggesting that these therapies may be appropriate for lung cancer patients that exhibit TUSC2 loss with concomitant alterations in EGFR or AKT activity [34,38,43,73]. 

In addition to the EGFR pathway, Deng et al. report that TUSC2 has increased anti-tumor effects with tumor suppressor p53 in lung cancer [78]. Co-expression of TUSC2 and p53 via nanoparticle-mediated gene transfer more significantly inhibited NSCLC growth and induced more apoptosis than treatment with either tumor suppressor alone in vitro. These findings were supported in vivo in which co-overexpression of TUSC2 and p53 significantly suppressed orthotopic NSCLC tumor growth in mice. Furthermore, TUSC2 significantly decreased expression of murine double minute-2 (MDM2), the E3 ligase responsible for mediating p53 proteasomal degradation, and increased activation of apoptotic protease-activating factor 1 (Apaf-1), promoting TUSC2 and p53 mediated tumor suppression [78]. TUSC2 downregulation of MDM2 increases p53 expression and promotes Apaf-1 activation, which enhances NSCLC chemosensitivity to cisplatin treatment, resulting in greater inhibition of tumor growth and increased apoptosis [79]. Interestingly, mass spectrometry analysis found protein–protein interaction between TUSC2 and Apaf-1. However, the importance of this interaction has yet to be elucidated [80]. Overall, TUSC2 is an established tumor suppressor frequently lost in lung cancer patients and has promising anti-tumor effects in both in vitro and in vivo studies.

### 3.2. Breast Cancer

Breast cancer is the most commonly diagnosed cancer in women and is the second leading cause of female related cancer deaths [56]. Over 300,000 cases and nearly 44,000 deaths are estimated in females in the United States in 2023 [56]. Breast cancers are categorized into four molecular subtypes, consisting of Luminal A, Luminal B, HER2-enriched, and triple-negative breast cancer (TNBC) [81]. Luminal subtypes are the most frequently diagnosed breast cancer subtypes with the most favorable treatment outcome, whereas the HER2-enriched and TNBC subtypes have worse clinical outcomes with higher propensity for metastases [81,82]. Patients with luminal subtypes of breast cancer are treated with hormone therapy, with neoadjuvant chemotherapy also being used to treat the more aggressive luminal B subtype [82]. HER2-enriched breast cancer patients are treated with anti-HER2 antibodies, such as trastuzumab (Herceptin). However, resistance to the anti-HER2 treatment is common, and it is often supplemented with neo-adjuvant chemotherapy treatment [82]. Due to the absence of ER/PR/HER2 receptors, TNBC is more difficult to treat and therapeutic options are limited [82].

In addition to being found in lung cancers, chromosome 3p abnormalities frequently occur in breast cancer patients, specifically within region 3p21.3, and suggests that TUSC2 is frequently lost in breast cancer (Figure 2) [1,7,83]. TUSC2 protein expression is significantly decreased in breast cancer tissues compared to normal breast tissue. However, no promoter methylation was found in the breast cancer tissues examined [84,85]. Moreover, de Costa Prando et al. found that TUSC2 expression in breast cancer was positively correlated with expression of *Ras association domain family member 1 A* (*RASSF1A*), which flanks the 3p21.3 chromosomal region. *RASSF1A* was found to be heavily epigenetically regulated and frequently suppressed in breast cancer. Additionally, a significant decrease in *RASSF1A* expression was correlated with an overall decrease in TUSC2 expression and other 3p21.3 TSGs, suggesting that epigenetic silencing of genes flanking the 3p21.3 region may lead to repression of TUSC2 and TSGs in this gene region. Conversely, upregulation of *RASSF1A* through promoter de-methylation increased the expression of *RASSF1A*, TUSC2, and other 3p21.3 associated TSGs [84]. 

In addition to TUSC2 loss via deletion or silencing of the 3p21.3 chromosomal region, TUSC2 expression is regulated by miR-138 in breast cancer [20]. MiR-138 is significantly upregulated in breast tumor tissues and is inversely correlated with TUSC2 expression [20]. MiR-138 specifically binds to the 5′-UTR of TUSC2 within the translation start site, silencing TUSC2 protein translation in TNBC [20]. Silencing of miR-138 led to a significant increase in TUSC2 expression, promoted TNBC apoptosis, and prevented tumor growth in vivo [20]. Although TUSC2 is downregulated by miRNAs, two TUSC2 pseudogenes (TUSC2P), identified on the X and Y chromosomes, act as a miRNA sponge to prevent miRNA targeting of TUSC2 [19,67,69,85]. TUSC2P shares 89% homology with the TUSC2 3′-UTR, and TUSC2P overexpression in breast cancer cells resulted in decreased proliferation, colony formation, survival, migration, and invasion of breast cancer mediated by TUSC2 [85]. TUSC2P has been shown to sequester multiple miRNAs, such as miR-661, miR-229-3p, miR-93, miR-17, miR-608, and miR-502, ultimately resulting in increased TUSC2 expression in multiple breast cancer lines [86]. Together, the literature establishes TUSC2 as a candidate breast cancer tumor suppressor frequently lost in breast cancer due to 3p21.3 chromosomal region deletion and silencing or via the targeting of TUSC2 mRNA by miR-138. 

### 3.3. Glioma

Gliomas are the most common brain tumors diagnosed in adults and often result in poor patient survival [87]. Gliomas are classified into one of four glioma grades [88,89,90]. Grades I and II are classified as non-malignant, whereas grades III and IV (GBM) are malignant gliomas, with grade IV being the most common and aggressive of all four gliomas [89]. Overall patient survival decreases with increasing grades of glioma. However, overall survival time for each grade varies depending on the genetic profile of each glioma [91]. The current standard of care for glioma patients consists of complete tumor resection, followed by radiation therapy and treatment with chemotherapy, the most common of which is temozolomide [92].

TUSC2 loss was first identified in gliomas by Xin et al. in 2015, who found that TUSC2 protein expression was significantly decreased in higher grades of glioma when compared to normal brain tissues, with grade IV GBM having the lowest TUSC2 expression (Figure 2) [93]. Additionally, lower TUSC2 expression is predictive of worse overall survival in glioma and GBM patients [11,93]. TUSC2 expression is regulated by multiple mechanisms in gliomas, including regulation via miRNA and circular RNAs, as well as regulation by post-translational modifications [11,94,95]. MiR-183-5p is significantly upregulated in glioma samples and was found to specifically target the TUSC2 3′UTR, predicted using starBase, which results in inhibition of TUSC2 mRNA translation and TUSC2-mediated glioma suppression [94,96,97]. Interestingly, a tumor suppressive circular RNA, circ-EGFR, antagonizes miR-183-5p and act as a sponge to sequester miR-183-5p and ultimately inhibits glioma progression through rescuing TUSC2 expression [94]. Although both circ-EGFR and TUSC2 protein expression are low in glioma samples, overexpression of circ-EGFR significantly increased TUSC2 protein expression and promoted TUSC2-mediated inhibition of glioma proliferation, migration and survival in vitro and in vivo [94]. Moreover, overexpression of circ-EGFR led to increased apoptosis, decreased proliferation, migration and invasion of glioma cell lines through inhibition of miR-183-5p and ultimately the restoration of TUSC2 expression [94]. Another miRNA, miR-106b-5p, was found to be significantly upregulated in gliomas and suppresses TUSC2 expression [95,98]. Similarly to circ-EGFR, a long non-coding RNA, GAS5-AS1, was found to bind and sequester miR-106b-5p to antagonize miR-106b-5p mediated TUSC2 suppression [95]. GAS5-AS1 overexpression resulted in a significant increase in TUSC2 and promoted glioma suppression in vitro [95]. Together, these studies demonstrate the importance of TUSC2 post-transcriptional regulation on promoting glioma progression.

TUSC2 is also regulated post-translationally in GBM via the proteasomal degradation system [11]. We found TUSC2 protein to have significantly decreased protein stability in GBM compared to normal human astrocytes. However, the addition of proteasome inhibitor MG132 stabilizes TUSC2 protein, indicating that loss of TUSC2 expression is critically mediated by the proteasomal degradation pathway. We recently demonstrated that TUSC2 protein stability in GBM is regulated by the E3 ubiquitin ligase Neural precursor cell expressed developmentally down-regulated protein 4 (NEDD4). We found that NEDD4 is overexpressed in GBM and that NEDD4 protein expression is inversely correlated with TUSC2 protein expression in GBM patient samples. We demonstrated that NEDD4 targets TUSC2 residues K71, K84, and K93 for poly-ubiquitination, and NEDD4 mediated TUSC2-K71 poly-ubiquitination promotes TUSC2 proteasomal degradation [11].

Although TUSC2 is frequently lost in gliomas, TUSC2 overexpression in glioma cell lines has been shown to decrease cell proliferation, migration, and invasion, and increase apoptosis in vitro [11,93,94]. Additionally, following the establishment of our orthotopic GBM mouse tumor model, we found that induction of TUSC2 expression resulted in a significant reduction in tumor growth, improved overall survival, and greater tumor cell apoptosis, demonstrating TUSC2 mediated tumor suppression in GBM in vivo [11]. One potential mechanism of TUSC2 mediated GBM tumor suppression may be through the regulation of the anti-apoptotic factor Bcl-xL [11]. Our RNA-seq analysis of a GBM TUSC2-KO cell line revealed over 1200 differentially expressed genes in GBM, with Bcl-xL, a critical component in preventing intrinsic apoptosis in cancer cells, being one of the genes significantly upregulated upon TUSC2-KO. We found that TUSC2 loss led to a significant increase in Bcl-xL expression, whereas TUSC2 overexpression led to a significant decrease in both Bcl-xL mRNA and protein expression in GBM. In our control and TUSC2-KO GBM cell lines, treatment with the Bcl-xL specific BH3 mimetic A-1331852 led to a significant increase in apoptosis only in TUSC2 positive cells, indicating an important connection between TUSC2-mediated apoptosis and Bcl-xL activity in GBM [11]. Another possible mechanism of TUSC2 mediated glioma tumor suppression may be through TUSC2 upregulation of miR-197 [93]. Interestingly, miR-197 expression is significantly downregulated in gliomas and is positively upregulated upon TUSC2 protein expression [93]. Upregulation of miR-197 resulted in glioma growth inhibition, while inhibition of miR-197 attenuated TUSC2-mediated glioma tumor suppression [93]. Overall, the current findings strongly suggest that TUSC2 is a novel glioma tumor suppressor that is frequently lost in gliomas due to miRNA-mediated downregulation and proteasomal degradation, with TUSC2 overexpression leading to glioma suppression. 

### 3.4. TUSC2 in Other Cancers

Since the discovery of TUSC2 as a candidate tumor suppressor frequently lost in lung cancer, TUSC2 has been found to be lost in many other cancer types, which further supports TUSC2 as an important tumor suppressor. This section will discuss TUSC2 loss and its putative functions in other cancer types.

TUSC2 expression is significantly decreased in both anaplastic thyroid carcinoma (ATC) and papillary thyroid cancer cells (PTC) compared to normal thyroid samples [18,99]. This decrease in TUSC2 expression is mediated by miR-584, which is significantly upregulated by the transcription factor TWIST, in thyroid cancer, discovered by Orlandella and colleagues. Overexpression of miR-584 in TPC-1 papillary thyroid cancer cells significantly decreases apoptosis and significantly decreases TUSC2 mRNA and protein expression. However, inhibition of miR-584 restored TUSC2 expression and led to enhanced thyroid cancer cell apoptosis [99]. Furthermore, Mariniello et al. found that overexpression of TUSC2 in both ATC and PTC cell lines resulted in decreased proliferation, migration, and invasion, as well as increased apoptosis, and these phenotypes were reversed upon deletion of TUSC2. Additionally, TUSC2 was found to induce thyroid cancer cell apoptosis via intrinsic apoptosis factors SMAC/DIABLO and cytochrome C upregulation. Thyroid cancer cell lines overexpressing TUSC2 demonstrate a significant increase in SMAC/DIABLO and cytochrome C expression following treatment with an apoptosis inducer, suggesting that TUSC2 sensitizes thyroid cancer cells to apoptosis via upregulation of SMAC/DIABLO and cytochrome C [18]. 

In ovarian cancer samples, Xie et al. found TUSC2 mRNA and protein expression to be significantly decreased as compared to normal patient tissue samples. TUSC2 mRNA downregulation in ovarian cancer is mediated by miR-663 interaction with the TUSC2 3′-UTR. MiR-663 is significantly up-regulated in both ovarian cancer cell lines and patient tumor samples. Overexpression of miR-663 promoted ovarian cancer migration and invasion and cellular proliferation [19]. Additionally, miR-663 was also found to target TUSC2 and to be significantly upregulated in bladder cancer [100]. Inhibition of miR-663 resulted in decreased bladder cancer cell viability, and increased expression of p53 and p21. However, knock-down of TUSC2 abrogated the effects of miR-663 inhibition [100]. These results demonstrate the importance of TUSC2 downregulation via miR-663 in promoting ovarian and bladder cancer progression [19,100]. 

In esophageal squamous cell carcinoma (ESCC), Liu et al. found TUSC2 mRNA expression to be significantly decreased in patient tumor samples compared to adjacent normal tissue [101]. Additionally, they found patients with low TUSC2 mRNA expression to have significantly worse overall survival. This study also illustrated that TUSC2 mRNA downregulation is mediated by multiple miRNAs, including miR-17-5p, miR-520a-3p, miR-608, and miR-661 in ESCC. However, overexpression of the TUSC2 pseudogene, TUSC2P, resulted in a significant increase in TUSC2 protein expression and a subsequent decrease in ESCC proliferation and invasion in vitro. TUSC2P was found to bind and sequester miR-17-5p, miR-520a-3p, miR-608, and miR-661 to prevent binding to the TUSC2-3′UTR, increasing TUSC2 expression in ESCC cells in vitro [101].

In head and neck cancers, TUSC2 is partially methylated in cancerous tissue. However only 0.1% TUSC2 methylation was detected in normal mucosa and nearly no methylation was detected in salivary gland rinses [102]. Due to the drastic differences in TUSC2 methylation status between normal and head and neck cancer tissues, TUSC2 is suggested as a potential biomarker for head and neck cancer initiation [102]. However, further investigation is necessary to establish TUSC2 methylation as a potential biomarker in head and neck cancers.

TUSC2 is also significantly decreased in acute myeloid leukemia (AML). Patients with low TUSC2 expression have a worse prognosis, shorter overall survival, and shorter leukemia-free survival [103]. MiR-378 has previously been shown to target TUSC2 and decrease TUSC2 mRNA and protein expression and is found to be significantly upregulated in AML patients [67,68,104,105]. Additionally, higher miR-378 expression is associated with worse AML patient survival [104]. Interestingly, miR-378 expression is negatively correlated with TUSC2 expression in AML and may potentially promote AML progression through downregulation of TUSC2 [103].

## 4. TUSC2 Cancer Therapy

Current anti-cancer drug therapies are primarily aimed at inhibiting target oncogenes to impede cancer growth and progression. However, therapies that aim to restore expression or replace function of lost tumor suppressors are under-developed. Designing treatments to either increase tumor suppressor protein expression, or to replace the tumor suppressor function is especially challenging with conventional drug discoveries and current therapeutics in cancer. Nevertheless, restoration of tumor suppressor expression through targeted nanoparticle delivery systems has shown promise as a potential therapeutic strategy, as seen with TUSC2 and lung cancer [106]. In 2012, a Phase I clinical trial (NCT00059605) was conducted on 31 recurrent or metastatic lung cancer patients previously treated with platinum based chemotherapy, in which they were treated with *N*-[1-(2,3-dioleoyloxy)propyl]-*N*,*N*,*N*-trimethylammonium chloride (DOTAP):cholesterol nanoparticles containing a TUSC2 expression plasmid [106]. Patients were treated intravenously (IV) every three weeks with one of six doses (ranging from 0.01 to 0.09 mg/kg) of the TUSC2 carrying nanoparticle, showing a maximum tolerated dose of 0.06 mg/kg. The trial resulted in five patients achieving stable disease, as well as a large portion of patients successfully having increased TUSC2 mRNA and protein expression in post-treatment biopsy samples. Additionally, multiple apoptotic proteins involved in the intrinsic apoptosis pathway were significantly upregulated in patients post-treatment. The results from this trial demonstrated that treatments with nanoparticles carrying a TUSC2 expression plasmid are able to be safely administered through IV treatment and result in increased expression of the tumor suppressor TUSC2, as well as upregulation of pro-apoptotic genes in recurrent and metastatic lung cancer patients, with the potential of stabilizing disease progression [106]. In fact, a clinical trial is currently in progress using the TUSC2 expression plasmid containing DOTAP:cholesterol nanoparticles (REQORSA) in combination with EGFR inhibitor Osimertinib (NCT04486833) in advanced lung cancer patients who experienced disease progression on Osimertinib alone [107]. The previous and current clinical trial are providing the necessary groundwork to enhance targeted cancer therapy utilizing tumor suppressor genes.

## 5. Conclusions, Open Questions, and Future Directions

Since the discovery of TUSC2 in 2000, TUSC2 has been shown to play many regulatory and tumor-suppressive roles in normal and cancerous tissues. TUSC2 has been established as an important regulator in the innate immune response and NK cell maturation. Additionally, TUSC2 mitochondrial functions are critically important for proper T cell response, regulation of ROS, as well as overall cellular mitochondrial calcium regulation and homeostasis. TUSC2 loss is linked to aging and the formation of sAD and premature hearing loss following unregulated oxidative stress in cells lacking TUSC2 expression. Along with the multiple roles TUSC2 plays in the normal cell population, TUSC2 also functions as a tumor suppressor in multiple cancer types. TUSC2 expression is commonly lost in many cancers due to somatic deletion, transcriptional downregulation, post-transcriptional downregulation, and post-translational regulation via the proteasomal degradation system. However, TUSC2 restoration in these cancer types commonly results in decreased cell proliferation, increased apoptosis, and overall reduction in tumor growth. 

The number of studies focused on TUSC2 has drastically increased over the years. However, there are still many TUSC2 related open questions that have yet to be addressed. The protein structure of TUSC2 has not yet be solved, which presents a major challenge in delineating TUSC2′s potential functions. The availability of TUSC2′s tertiary structure would provide structural insight into the predicted functional domains of TUSC2. In addition to the confirmed N-terminal myristoylation at Glycine-2, TUSC2 amino acid sequence predicts a myristoylation binding pocket, as well as an EF-hand calcium binding domain. The TUSC2 protein sequence and predicted functional domains are most homologous to calcium myristoyl switch proteins, such as recoverin [14,33]. Therefore, solving the protein structure of TUSC2 would confirm if there is in fact a calcium binding pocket within the predicted EF-hand domain of TUSC2. Furthermore, investigating whether TUSC2 binds to calcium, and if the binding subsequently releases the N-terminal myristoylation to anchor TUSC2 in the mitochondrial membrane, is a necessary next step.

TUSC2 has been reported to be involved in altering functions of multiple proteins and malignant phenotypes, including, EGFR, AKT, AMPK, p53, apoptosis, cancer stem cells, immune response, and calcium homeostasis. TUSC2 has also been shown to alter the gene transcriptome. However, how TUSC2 alters gene expression, protein phosphorylation, and other cellular processes mentioned above is currently unknown. Filling these knowledge gaps is needed to further elucidate the developmental role of TUSC2 in normal tissue, as well as the mechanisms by which TUSC2 functions as a tumor suppressor. 

Discovering additional TUSC2-interacting proteins would further establish potential functions of TUSC2 in both normal and cancerous tissues. The putative mitochondrial functions of TUSC2 have been linked to mitochondrial calcium regulation, the innate immune response, calcium related gene regulation, and aging. However, the exact functions of mitochondrial TUSC2 remain unknown. TUSC2′s mitochondrial functions are primarily linked to mitochondrial calcium movement. Therefore, TUSC2 may interact with calcium influx/efflux proteins to modulate cellular calcium signaling. Identifying these potential interactions is critical in defining TUSC2′s functional impact on the various processes taking place in the mitochondria. Additionally, TUSC2 has previously been shown to interact with two proteins important for regulating cancer progression and apoptosis, c-Abl and Apaf-1, respectively. However, the importance of these interactions has yet to be elucidated [76,80]. This suggests that TUSC2 may interact with other oncogenes and tumor suppressors as potential mechanisms that underly TUSC2-mediated tumor suppression across multiple different cancer types. 

Furthermore, in both normal and cancerous cells, TUSC2 has been shown to play indirect roles in transcriptional regulation. Evidence suggests this may be through altering cellular calcium levels, as seen by TUSC2 regulation of calcium-dependent transcription factors NFAT and NF-κB [46]. However, it may also be through direct interaction with transcription factors, which has not been explored. Further investigating the role of TUSC2 in calcium regulation, as well as the potential interaction with transcription factors, would better define the role and mechanism of TUSC2 in transcriptional regulation.

Although TUSC2 elicits tumor suppressive effects, the role of TUSC2 loss in tumorigenesis remains uninvestigated and warrants further research. Several studies have shown that conventional TUSC2-KO mice undergo changes in the immune response, aging, and mitochondrial homeostasis [43,52,53]. To conclusively demonstrate the tumorigenic role of TUSC2, tissue-specific conditional deletion of TUSC2 would be needed for elucidating the oncogenic phenotypes that arise upon TUSC2 loss. Therefore, developing a conditional Cre recombinase-mediated TUSC2-KO mouse model is critical to study how tissue-specific TUSC2 loss promotes spontaneous tumorigenesis. 

Due to the prevalence of TUSC2 loss in many cancer types, investigations into therapeutic modalities that restore TUSC2 expression is a major topic of research. Rescuing TUSC2 expression through TUSC2 vector-carrying nanoparticles (REQORSA) treatment has demonstrated positive results in lung cancer patients [106]. Although multiple patients did show stable disease, as well as successful upregulation of TUSC2 protein expression in lung cancer biopsies, the majority of the patients had no response. Current efforts are focused on a second clinical trial using REQORSA in combination with EGFR inhibitor Osimertinib (NCT04486833). Additional insights into the mechanism for tumor resistance to TUSC2 restoration would be important to improve patient response. Since TUSC2 expression is inhibited by miRNAs across multiple cancer types, developing a nanoparticle carrying a TUSC2P expression vector may result in increased TUSC2 protein expression through sequestration of the TUSC2-targeting miRNAs. Alternatively, identifying therapeutic agents that directly bind TUSC2 and promote TUSC2 protein stability, or that target newly discovered TUSC2-interacting proteins, could be another viable treatment method to explore.
